# Biological surface properties in extracellular vesicles and their effect on cargo proteins

**DOI:** 10.1038/s41598-019-47598-3

**Published:** 2019-09-10

**Authors:** Laura Santucci, Maurizio Bruschi, Genny Del Zotto, Francesca Antonini, Gian Marco Ghiggeri, Isabella Panfoli, Giovanni Candiano

**Affiliations:** 1 Laboratory of Molecular Nephrology, IRCCS Istituto Giannina Gaslini, Genoa, Italy; 2 Department of Research and Diagnostics, IRCCS Istituto Giannina Gaslini, Genoa, Italy; 3 Division of Nephrology, Dialysis, and Transplantation, Scientific Institute for Research and Health Care, IRCCS Istituto Giannina Gaslini, Genoa, Italy; 4 Department of Pharmacy-DIFAR, University of Genoa, Genoa, Italy

**Keywords:** Biological techniques, Isolation, separation and purification

## Abstract

Ultracentrifugationon sucrose density gradientappears to be the best purification protocol for extracellular vesicle (EVs) purification. After this step, to reduce disulfide bridges linking exogenous proteins to the vesicles, the collected samples are routinely washed and treated with dithiothreitol (DTT). Such incubations are performed at temperatures ranging from room temperature up to 95 °C, with either Tris or PBS as buffers. We re-investigated these steps on both exosomes and microvesicles purified from blood (serum) and urine by electrophoretic separation, silver staining and western blots analysis. Data confirm that an extra centrifugation on a sucrose cushion can effectively eliminate contaminants. Tris buffer (50 Mm) and β-mercaptoethanol as a reducing agent at room temperature dramatically improved either sample cleaning. By contrast, especially for exosomes PBS buffer and DTT, above 37 °C, caused massive protein aggregations, yielding blurred SDS-PAGE gels in both samples. Immuno-blot analyses demonstrated that in PBS-DTT contamination with albumin (in serum) or with uromodulin (in urine) occurs. DTT, likely due to its two–SH groups, might form scrambled SS-bonds promoting EVs interaction with environmental macromolecules via disulphide bridges. Therefore, to obtain maximum vesicle purity for biomarker investigations and to maximize both presence of EVs proteins and their accessibility, use of DTT is not recommended.

## Introduction

Extracellular membrane vesicles (EVs) from biological fluids have been proposed as a promising source of protein biomarkers^1^. Microvesicles (MVs, 100–1,000 nm)^2^ and exosomes (EXOs, 30–100 nm)^3^ are shed by all mammalian cells, including malignant ones. EVs can transfer proteins, various RNA forms, and other cellular components contributing to intercellular communication network^4^. Currently considered as a potential reservoir of protein markers, EVs have the advantage to circumvent the challenge represented by sera, where the dynamic protein concentration range can span up to 10–12 orders of magnitude. The active role of exosomes in cancer biology has been established. Cancer cells secrete EVs implied in cancer growth and metastasis that have a potential for biomarker identification^5^. We have previously shown that most of urinary proteome contained in the EXOs can be effectively utilized to study the whole urinary tract^6^. Also, urinary EXOs possess oncogenic properties^7^. In a recent report, investigations on urinary EXOs resulted in the detection of few antigens that, with high specificity and sensitivity, directly correlated with alterations in prostate cancer tissues, allowed a better patient stratification^8^. We have also shown that both MV sand EXOs conduct aerobic metabolism^9^. Such metabolic ability of EVs bears a clinical relevance, especially for MVs that seem to more closely reflect the cell of origin, as they originate from the plasma membrane. This opens the perspective to utilize MVs as sensors of the metabolic state of the organism, especially in the case of the premature newborns^10^.

Although the analysis of EVs, especially those derived from blood, appears highly promising in for an early-stage diagnosis the field of precision medicine, from a technological point of view the purification method is quite laborious and presents potential pitfalls. In fact, especially when EVs are separated from biological fluids, the presence of other dimensionally similar “bodies”^11^ can represent an obstacle to their utilization. Several purification methods have been reported. Some of them take advantage of precipitation with salts (such as ammonium sulphate), polyethylene glycol (PEG) or organic solvents^12^. Others suggest purification via filtration using different types of membranes, bind-elute size exclusion chromatography or even capture with specific antibodies^3,13–19^. Many of these methods are currently under discussion: while ultracentrifugation is the most aknowledged^20^ method, the best seem to be those utilizing a sucrose density gradient, or a density cushion of known sucrose concentration that can significantly reduce the contaminant as abundant proteinacious presence and lipoprotein^21–26^ particles.

The generally accepted protocol for MVs/EXOs purification also includes a washing step followed by an incubation with dithiothreitol (DTT), to reduce possible SS-bonds to minimize the binding of non-vesicular proteins to the EVs. Such incubations with DTT are generally performed at temperatures ranging from 37 °C up to 95 °C^27–30^. The use of different buffers is also recommended, the preferred ones being Tris^5,9,28,31,32^ or phosphate buffered saline (PBS)^33–35^. Even though several papers described different purification methods for EVs, only few of them attempted to assess the quality and pureness of the sample by performing an electrophoretic analysis conducing an in-depth study into the gel band patterns.

Here, after are-evaluation by electrophoretic analysis of the above-mentioned methods, we present novel data supporting the notion that to obtain both EXOs and MVs free of major non-vesicular contaminants, coming from bodily fluid abundant proteins such as albumin and uromodulin a particular strategy is needed^23,36–39^.

## Results

### Sampling and analyses

MV and EXO were isolated by centrifugation from either blood or urine (second morning urine) from twelve healthy fasting donors (6 males and 6 females). Each sample was divided in aliquots and the different analyses were performed (cytofluorimetric validation, dynamic light scattering analysis, gel electrophoresis staining and western blotting) according to the method detailed in Supplementary Figs S1 and S2.

Sample loading was optimized by loading the same volume (1 ml for both serum and plasma) for all samples regardless of the sample protein concentrations. For western blot, samples were aligned with β-actin. The protein content of each pellet after purification was also tested (see Methods).

**Figure 1:**
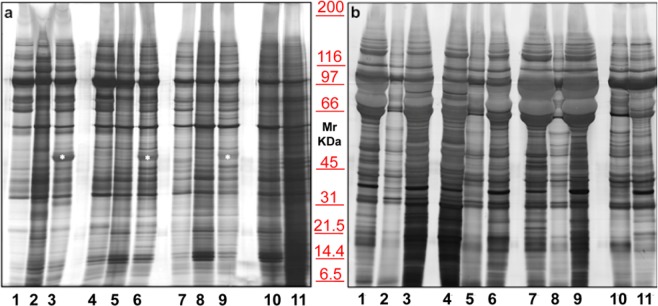
Representative electrophoresis of microvesicles (**a**) and exosomes (**b**) obtained from 1 ml of three different EDTA plasma (**1, 4, 7**), sera (**2, 5, 8**), sodium citrate plasma (**3, 6, 9**), serum pool (**10**) and EDTA plasma pool (**11**) of healthy donors. SDS-PAGE were performed onto 8–16% T gels. Both gels were visualized by silver staining.

To choose the best anticoagulant for blood collection, several tests were performed. Figure 1a shows the electrophoretic separation of three different sera (lanes **2, 5, 8**), compared to plasma samples treated with two different anticoagulants, namely: EDTA (lanes **1, 4, 7)** and sodium citrate (lanes **3, 6, 9**). The cleanest pattern was obtained using EDTA. This result is even more evident in EXOs **(Panel b)**. On the contrary, plasma treated with Sodium citrate tends to generate smearyruns (see bands in Fig. 1, in particular those highlighted with an asterisk), probably due to calcium bridges. A serum (lane **10**) and a plasma (lane **11**) pools were also run to confirm the results.

### Flow cytometry

**Figure 2:**
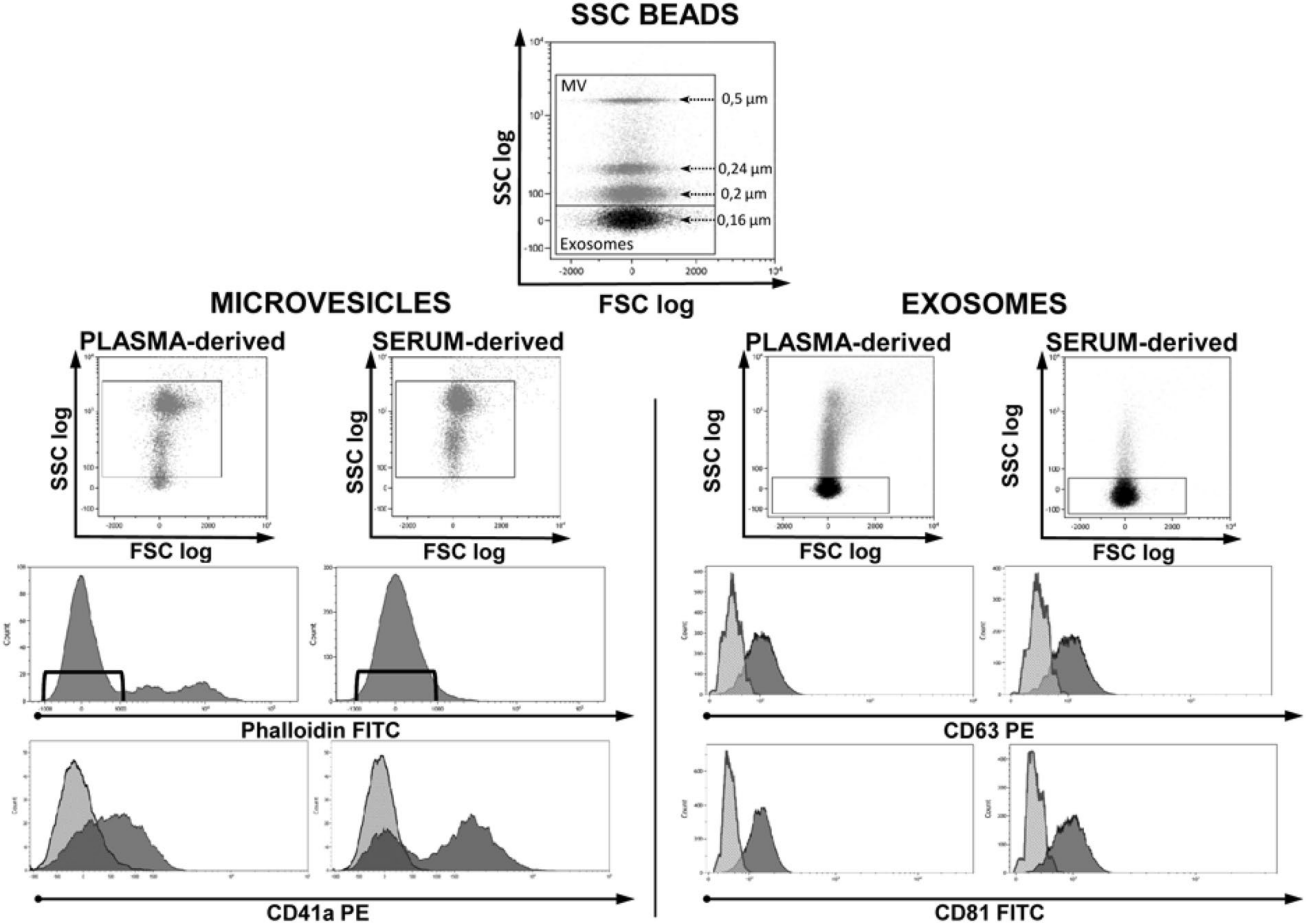
Cytofluorimetric analysis of microvesicles and exosomes obtained from plasma and serum of healthy donors. PB was collected in EDTA tubes. Top dot plot shows SSC beads analyzed by their physical parameters, namely side scatter (SSC) and forward scatter (FSC). Beads allowed to set gates used to define vesicles dimensions. Among events of microvesicular dimension only those negative for Phalloidin were considered MV and further analyzed for CD41a expression (left side dot plots). Considering the small dimensions of exosomes, to analyze their CD63 and CD81 expression (right side dot plots) a fluorescence minus one analysis was performed. CD41a, CD63 and CD81 histograms show both negative (light grey) and positive (dark grey) samples. The experiment is representative of other three.

As shown in Fig. 2 (left), MVs obtained from both serum and plasma were identified based on their dimensions and, to avoid debris, to their negativity to Phalloidin, a cyclic peptide that binds to f-actin with high affinity^40^. Then, to verify if they were compatible with platelet-derived MVs, samples were labeled with anti-CD41a.

The smaller micro-particles, gated on the basis of their physical dimensions, were identified as EXOs due to their expression of CD81 and CD63 (Fig. 2, right). When comparing MVs and EXOs physical parameters (forward scatter FSC vs. side scatter SSC) of serum and plasma, it is clear that serum samples have less smear and seem to be cleaner. Moreover, when analyzing MVs coming from the same donor for the expression of CD41a, in serum it is possible to discriminate two different population (CD41a^+^ and CD41a^−^) while information was less clear in plasma. These analyses support the idea that serum could be the best blood-derived fluid to understand the as yet unclear surface interaction phenomena characteristic of MVs or EXOs.

### Dynamic light scattering analysis

**Figure 3:**
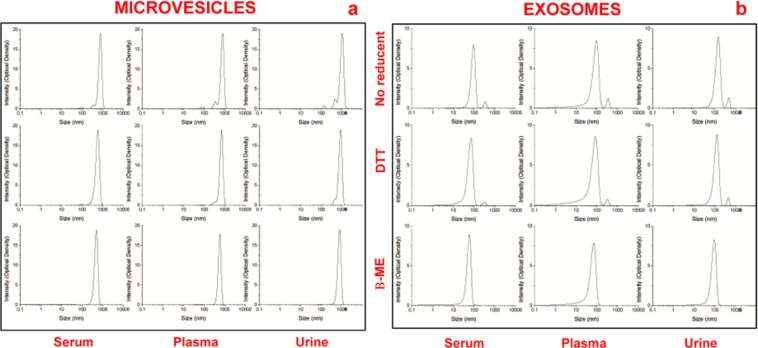
Representative dynamic light scattering analysis of microvesicles (**a**) and exosomes (**b**) obtained from 1 ml of serum/plasma and 12 ml of urine, after sucrose cushion. The graph reports the size (nm) of the particles (x-axis) versus intensity (Optical Density y-axis). The samples visualized in the first row are without reducing agents while those in the second and third row were treated with by DTT and β-ME, respectively.

Dynamic light scattering analysis was used to determine the size and purity of particles versus intensity (optical density). Results reveal a Gaussian distribution profile with a mean peak at 500 ± 65 nm or 50 ± 5 nm, the typical size for MVs or EXOs (Fig. 3), respectively. **Panel a** show the peaks of MVs and **panel b** of EXOs obtained from 1 ml of serum/plasma and 12 ml of urine, after sucrose cushion centrifugation. Samples visualized in the first lane are without reducing agent, while those in the second and third lanes were treated by DTT and β-mercaptoethanol (β-ME), respectively. Statistical difference between the sizes of MVs or EXOs (P < 0.05 for both types of extracellular vesicles) isolated using the different protocols was determined. Also, this analysis shows that the cleanest peaks were obtained for serum with respect to plasma that displays additional peaks. Also, the most suitable protocol for the three biological fluids was the one utilizing β-ME as a reducing agent, yielding narrower peaks throughout. Data confirm that serum is the cleanest sample, respect to plasma.

### Isolation and SDS-PAGE of serum extracellular vesicles

**Figure 4:**
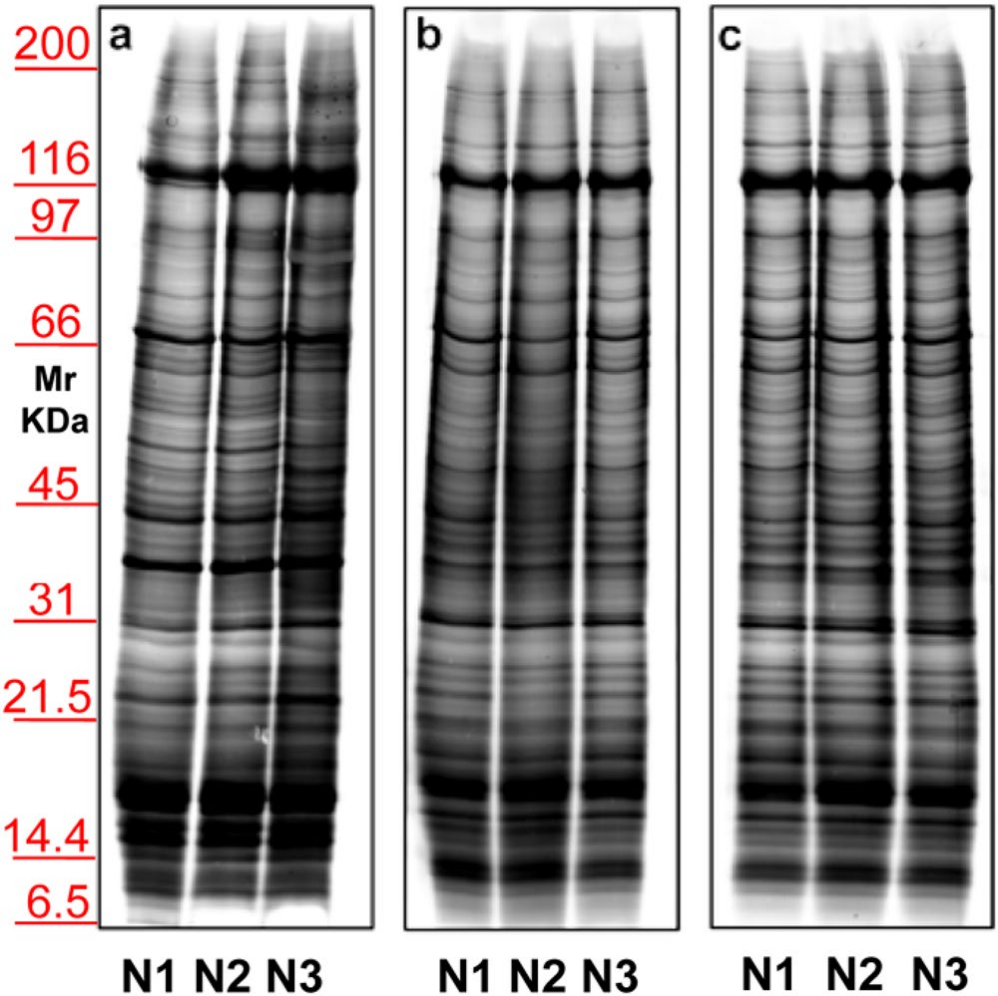
Representative electrophoresis of microvesicles obtained from 1 ml of three different sera (**N1–N3**) of healthy donors. Gels are cropped to better highlight the different methods used. Silver staining of samples without (**a**) or with (**b,c**) application of sucrose cushions. In panel b incubation was conducted at room temperature, while in (**c**) incubation was at 37 °C; in both cases the buffer used is Tris. SDS-PAGE was performed onto 8–16% T gels.

Considering the above-mentioned results, we decided to use exclusively serum, as blood-derived biological fluid. Samples were centrifuged twice, first to eliminate cell debris and subsequently to remove the microscopic particle sediments. Then, MVs and EXOs fractions (22,000 and 100,000 × g respectively) were purified. The same flow-chart (Supplementary Fig. S1) procedure was used for the analysis of the serum and urinary EVs. Then, electrophoresis of MVs proteins was performed on 1 mL of three different healthy donor sera (Fig. 4, **N1–N3**). **Panel a** shows a representative silver staining of serum proteins purified by centrifugation in the absence of a sucrose density cushion. **Panels b,c** show profiles of the same samples after an extra purification step by centrifugation on a sucrose cushion (Supplementary Fig. S2). This supplementary step was designed to eliminate contaminants in pellet preparation, such as non-specific proteins and large protein aggregates, which sediment by centrifugation but do not float on a sucrose cushion. In **Panel b** incubation was do neat room temperature while in **Panel c** at 37°Cfor 30 minutes. In both cases Tris was the buffer. The best result was obtained in the un heated sample after the application of sucrose cushions.

**Figure 5:**
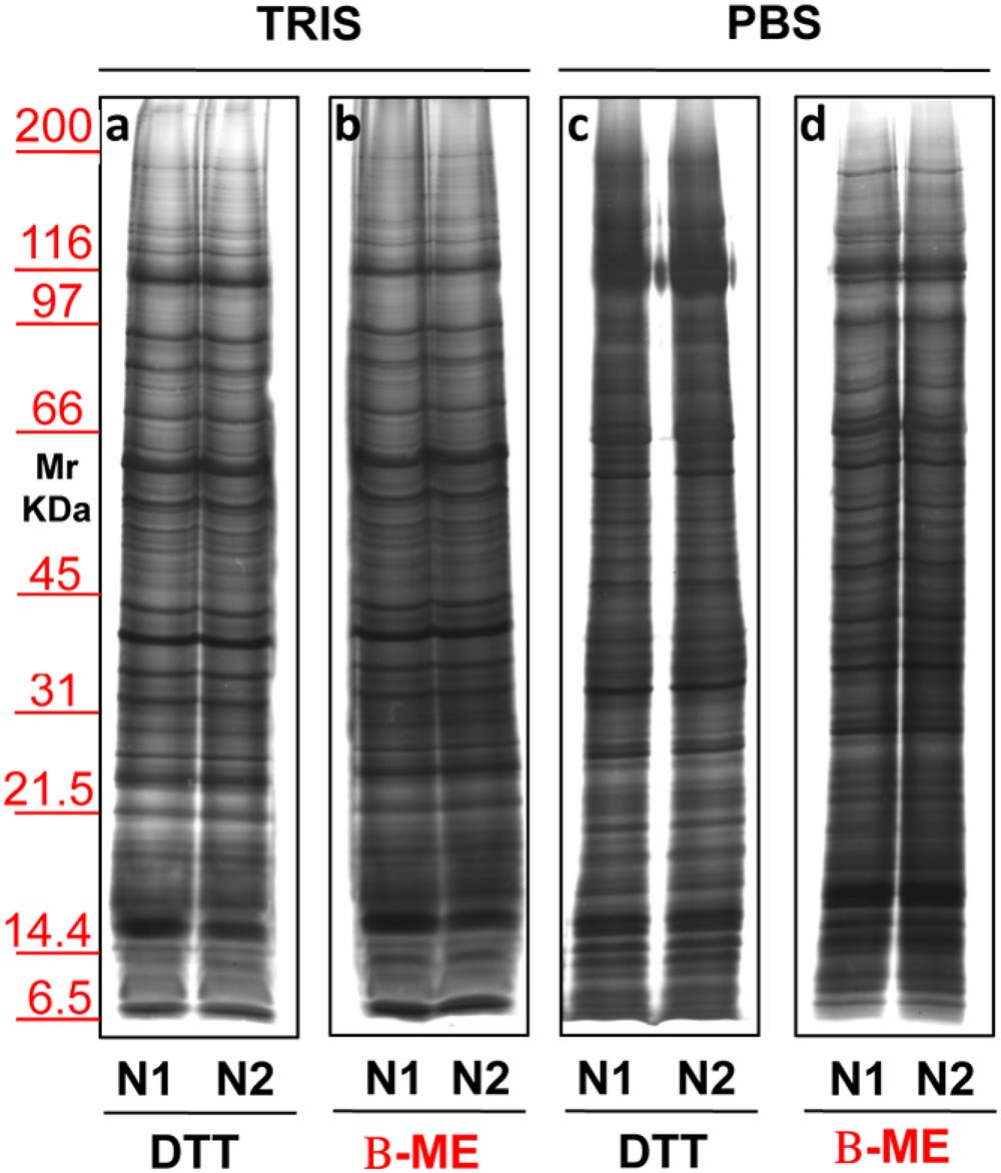
Representative electrophoresis of micro-vesicles obtained from 1 ml of two different sera (N1–N2) of healthy donors. Gels are cropped to better highlight the different methods used. Silver staining representative of samples isolated by sucrose cushion centrifugations utilizing as buffer Tris (**a,b**) or PBS (**c,d**). In panels a and c samples were added of DTT while those in panel b and d were added of β-ME. SDS-PAGE was performed onto 8–16% T gel.

Once we chose this condition, further procedure modifications (Fig. 5) were conducted on two different sera (**N1–N2**). Two different buffers, Tris (**Panels a**,**b**) or PBS (**Panels c**,**d**) were evaluated together with two different reducing agents, namely DTT (**Panels a**,**c)** or β-ME (**Panels b**,**d**). Results allowed to establish that the best sample cleaning was obtained using Tris as buffer with β-ME on the sucrose cushion.

**Figure 6:**
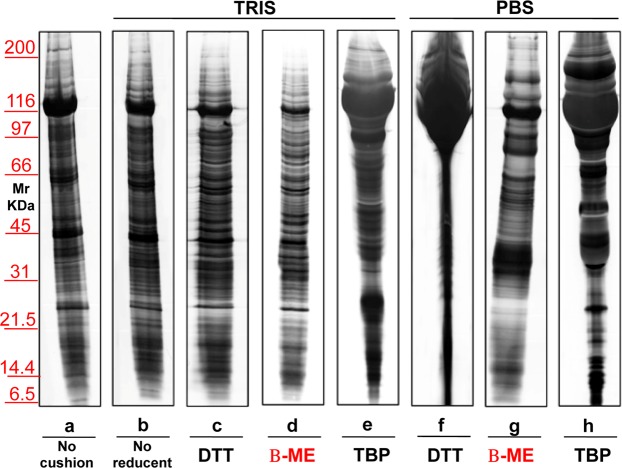
Representative silver staining of exosomes obtained from 1 ml of serum from healthy donors. Gels are cropped to better highlight the different methods used. Electrophoresis of samples isolated without (**a**) or with the use of sucrose cushions (**b**–**h**) utilizing as buffer Tris (**b**–**e**) or PBS (**f**–**h**). Samples visualized in lanes (**c**,**f**) were treated with the addition of DTT, those in (**d**,**g**) were added of β-ME, while those in (**e**,**h**) were added of TBP. Serum in (**b**) is without reducing agent. SDS-PAGE was performed onto 8–16% T gels.

Identical experiments were performed also on EXOs (Fig. 6) purified by ultracentrifugation without (lane **a**) or with a sucrose cushions (lanes **b–h**), using Tris (lanes **b–e**) or PBS (lanes **f–h**) as buffer. DTT was added to samples in lanes **c** and **f**, whereas β-ME in those in lanes **d** and **g**. No reducing agent was added to serum sample in lane **b**. Notably, DTT, in the presence of either Tris or PBS, causes a massive aggregation of proteins, likely due to its double –SH residues, which are absent using β-ME. Furthermore, the use of PBS in the presence of DTT worsened the run in EXOs when compared to MVs, the possible cause being the formation of ionic bridges between EXOs surface proteins and other extra-vesicular proteins, due to the reducing environment. A third agent, Tri-buthyl-Phosphine (TBP) which is a very strong reducing agent was also utilized in serum EXOs purification (see lanes **e**,**h)**. Interestingly, also TBP which does not contain –SH groups can create artifacts (see lanes **e**,**h**), likely promoting direct S-S bonding among SH-containing proteins, similarly to effect of DTT. The mechanism may be the initial strong reduction of the EXOs SH-containing proteins in the TBP-containing environment, over sucrose density cushions. Then, as soon as during ultracentrifugation the EXOs cross the cushions, a rapid random re-oxidation of -SH groups would occur.

Supplementary Fig. S3 shows the representative results of Tris buffer concentration titration (50 mM in **panel a**, 250 mM in **panel b** and 500 mM in **panels c**,**d**) on EXOs obtained from three different sera (**N1–N3**). 50 mM Tris appears the best buffer concentration, likely due toa “salting out” effect of high molar concentrations of Tris. Also for EXOs in the same conditions, the addition of β-ME (**Panel c**) produced the cleanest runs confirming the results obtained for MVs.

### Isolation and SDS-PAGE of urinary extracellular vesicles

**Figure 7:**
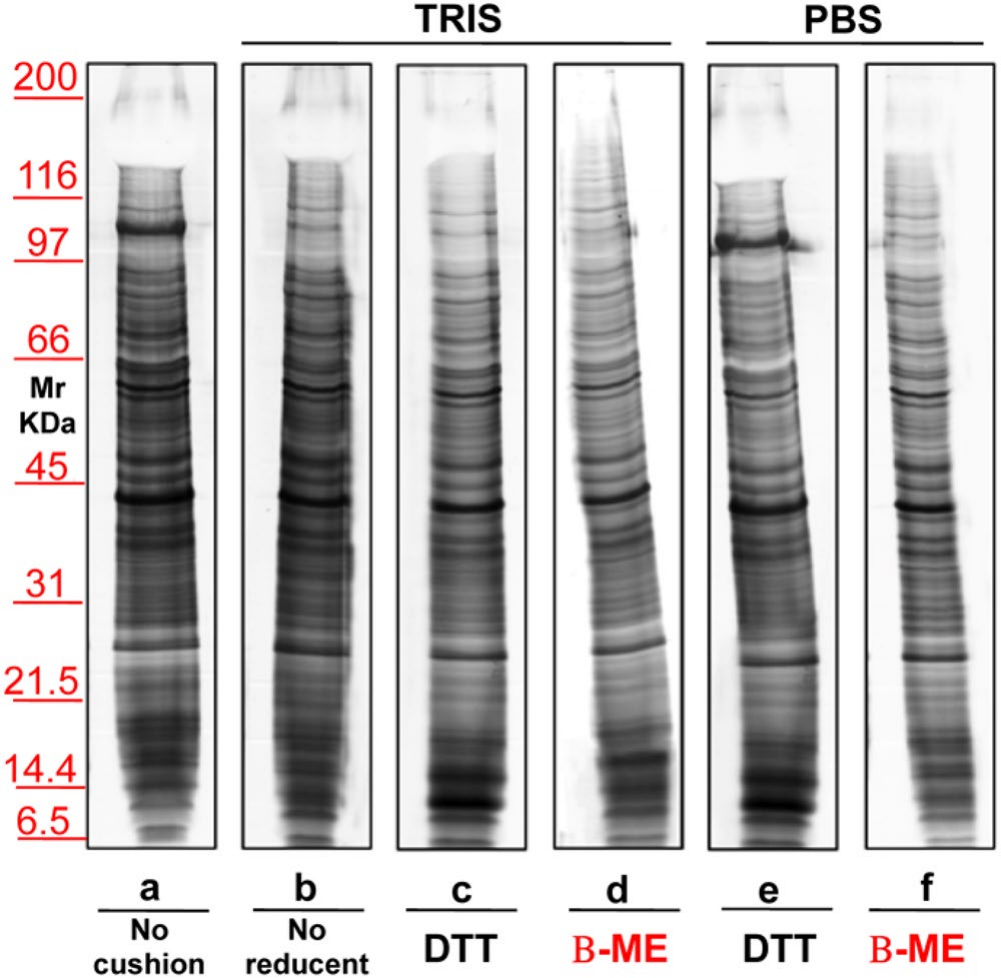
Representative silver staining of microvesicles obtained from 12 ml of urine of healthy donors. Gels are cropped to better highlight the different methods used. Electrophoresis without (**a**) and on a sucrose cushions (**b**–**h**) utilizing as buffer Tris (**b**–**e**) or PBS (**f**–**h**). The sample visualized in lanes (**c**,**f**) were added of DTT, those in (**d**,**g**) of β-ME while those in (**e**,**h**) of TBP. Serum in (**b**) is without reducing agent. SDS-PAGE was performed onto 8–16% T gels.

**Figure 8:**
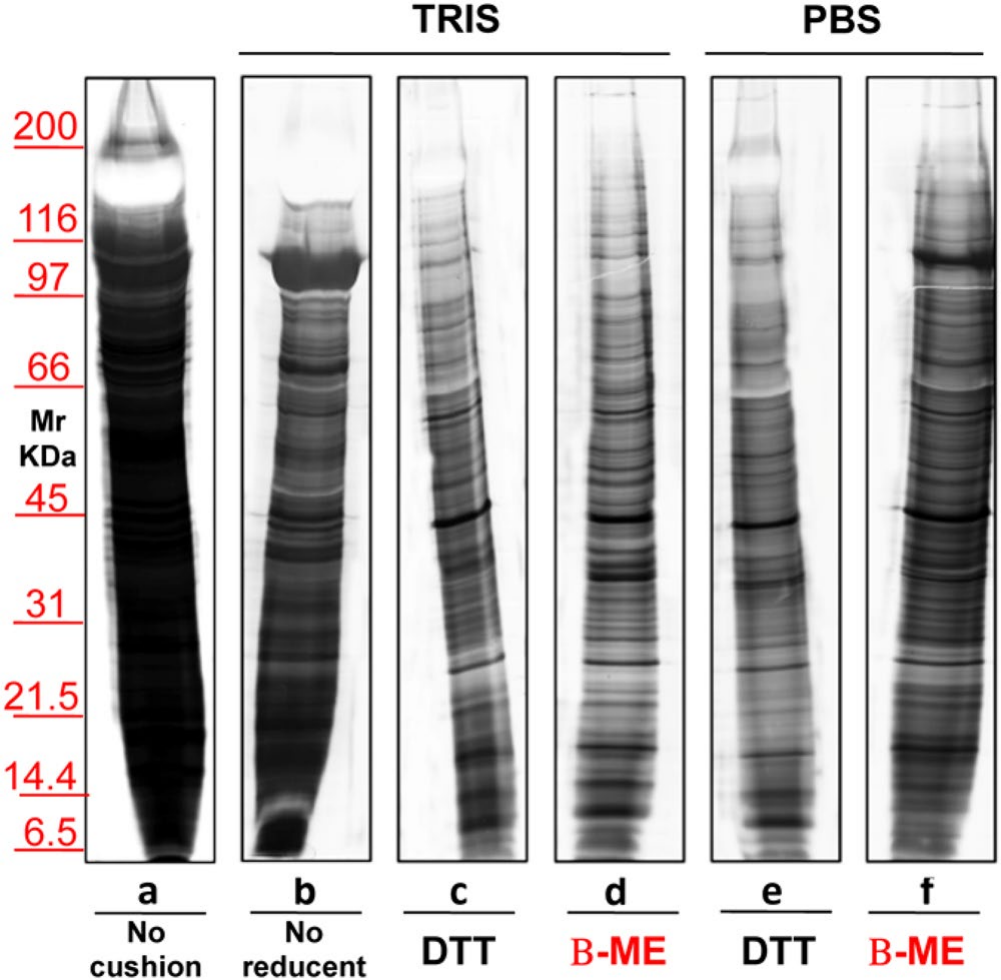
Representative silver staining of exosomes obtained from 12 ml of urine of normal volunteers. Gels are cropped to better highlight the different methods used. Electrophoresis without (**a**) and on a sucrose cushions (**b**–**f**) utilizing as buffer Tris (**b**–**d**) or PBS (**e**–**f**). The sample visualized in lane (**c**,**e**) is treated with the addition of reducent DTT while in panel d and f of β-ME. In the serum (**b**) there is no reducing agent. SDS-PAGE was performed onto 8–16% T gels.

The second biological fluid tested was urine. Figure 7 shows a representative silver staining of MVs proteins from 12 mL of urine of healthy donors, isolated without (lane **a**) or with a sucrose density cushion (lanes **b**–**f**) in the presence of either Tris (lanes **b–d**) or PBS (lanes **e,f**). Samples in lanes **c** and **e** were treated with DTT where as those in lanes **d** and **f** with β-ME. In lane **b** no reducing agent was added. These results confirm those obtained for serum, although in the case of urine the differences between the two buffers were less evident. The major protein that strongly decreases in presence of Tris and β-ME (lane **d**) is uromodulin, normally abundant in urines and particularly rich in cystein (visible as a negatively stained band in the upper gel region, at >100 KDa). Figure 8 shows the result of the protein separation of EXOs obtained from urine without (lane **a**) or with the use of a sucrose density cushion (lanes **b**–**f**) utilizing either Tris (lanes **b**–**d**) or PBS (lanes **e–f**), and with DTT (lanes **c**,**e**) or β-ME (lanes **d**,**f)**. In **b** there was no reducing agent. Again, addition of β-ME improved the separation in PBS, whereas DTT worsened the patterns in the presence of either buffer.

### Western blot analysis of serum and urinary extracellular vesicles

**Figure 9:**
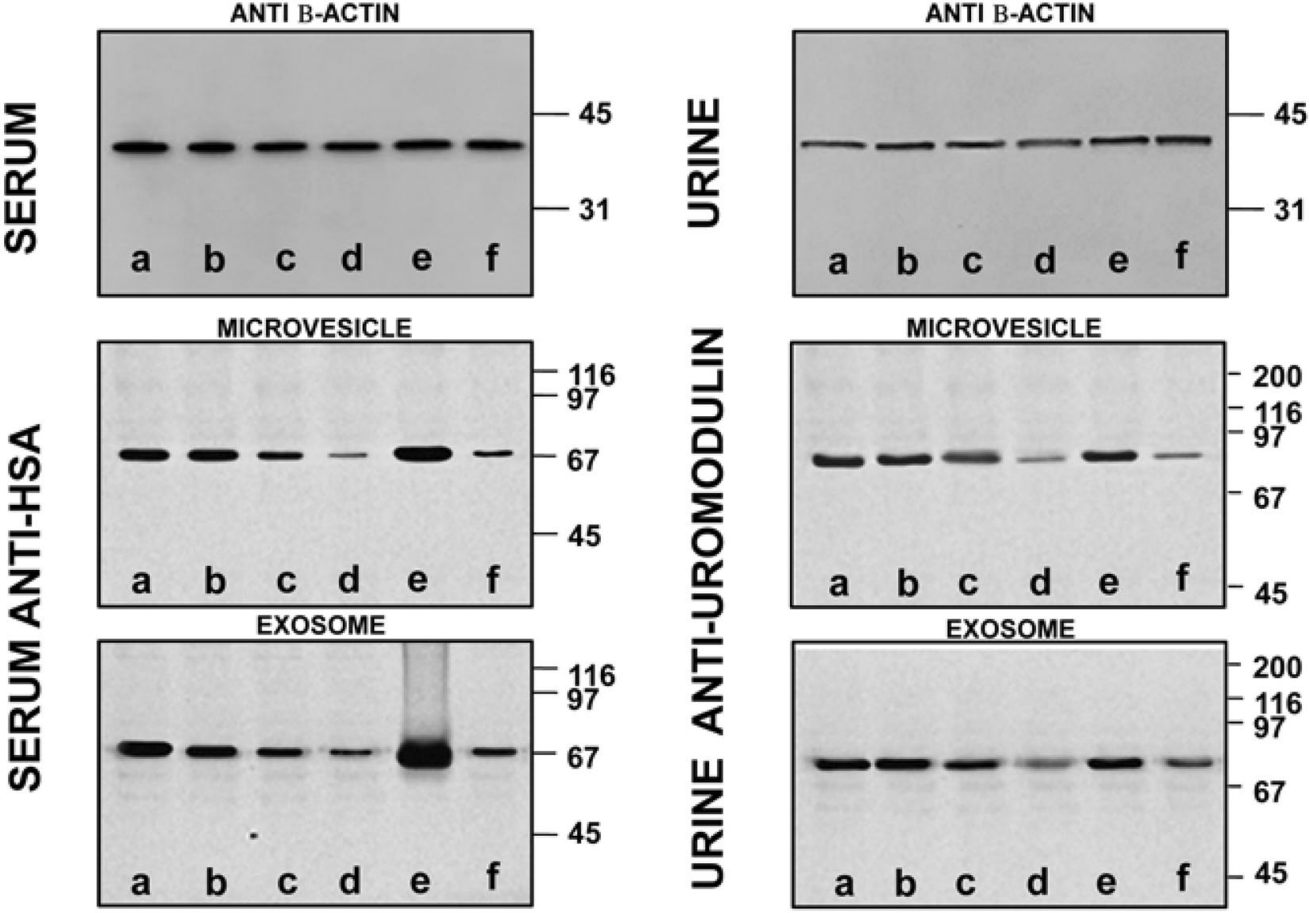
Western blot of MVs/EXOs of serum/urine from healthy donors with anti-human monoclonal mouse β-Actin, polyclonal rabbit serum anti-albumin (HSA) or sheep anti-uromodulin from samples isolated without (**a**) or with sucrose cushions (**b**–**f**) utilizing as buffer Tris (**b**–**d**) or PBS (**e,f**). Samples in (**c**,**e**) were added of DTT while those in (**d**,**f**) of β-ME. In the serum/urine (**b**) there is no reducing agent. Gels are cropped to better highlight the results. SDS-PAGE was performed onto 8–16% T gels.

The above results were confirmed by the experiments described in Fig. 9, showing results representative western blot of MVs and EXOs from serum and urine samples. As already mentioned, western blots were normalised by alignment of sample bands to β-actin. In this way it was possible to eliminate any artifact due to aggregated proteins. Then, polyclonal rabbit anti-human albumin (HSA) and polyclonal sheep anti-human uromodulin were used in samples treated without (lane **a**) or with sucrose density cushion (lanes **b–f**) by Tris (lanes **b–d**) or PBS (lanes **e–f**). Samples in **c** and **e** were treated with DTT whereas those in **d** and **f** with β-ME. In lane **b**, both for serum and urine, no reducing agent was added. Consistently to what already observed, the best conditions are obtained in presence of Tris-buffer plus β-ME, as confirmed in lane **d** by the markedly reduced levels of albumin and uromodulin as compared, for example, to lane **e**, where DTT was the reducing agent.

To further confirm the removal of non-vesicular contaminant proteins, we analyzed the serum supernatants from the washing steps after sucrose gradient. Supplementary Fig. 4 shows the supernatants from the first (MVs, lanes **1, 3, 5**; EXOs, lanes **7, 9, 11**) and the last (MVs, lanes **2, 4, 6**; EXOs, lanes **8, 10, 12**) wash. Polyclonal rabbit anti-human albumin (HSA) (chosen as a representative being among the most abundant proteins in the electrophoretic pattern) was used. In EXOs washings, albumin is more abundant, as expected since these vesicles yielded cleanest pellets after sucrose density gradient. Such result was less evident in the MV. In the respective pellets we studied the presence of apolipoprotein A1 (Apo-A1). This protein is a major component of HDL co-sediment with EXOs^41^. On the same gel, after membrane stripping, we also detected apolipoprotein E (Apo-E) and C (Apo-C). Supplementary Fig. 5 is a representative western blot with anti-human Apo-A1, Apo-E or Apo-C of EXOs pellets isolated after sucrose density gradient treated without (lanes **1, 4, 7**), or with reducing agents (DTT, lanes **2, 5, 8** or β-ME, lanes **3, 6, 9**). Anti β-actin was used as loading control. Data show that the sample treated with β-ME is the cleanest, with respect to the presence of HDL, consistently with previous results. By contrast, the absence of both ApoE and ApoC, rule out the presence of chylomicrons and VLDL, respectively.

## Discussion

EVs are constitutively released by cells into the extracellular environment^42^ and are present in all body fluids. EVs have gained widespread interest due to their ability to carry bioactive components such as RNAs, DNA, and proteins. However, besides their luminal cargo, EVs can also carry a significant surface cargo encompassing DNA and especially proteins likely acquired in body fluids after shedding^43^. Being especially true in the case of blood, this renders technically challenging the investigation of the EVs. Little is known about the purification conditions that favour the external adsorption of extravesicular proteins onto EVs or their loss. Moreover, most studies have considered EVs as a whole and did not distinguish EXOs from MVs. By contrast, our investigation focused on EXOs and MVs singularly. In particular, the present study involved an electrophoretic approach to study the inherent heterogeneity of the surface cargo of EXOs and MVs, depending on the purification conditions and the kind of EVs.

Although a combination of ultracentrifugation and size-exclusion chromatography (SEC) is often used, the gold-standard procedure for EVs purification is ultracentrifugation on a sucrose density gradient (or on a sucrose cushion), followed by washing steps and a final treatment with DTT, meant to reduce possible SS-bonds among EVs^13,21,30^. However, the content of surface thiols can also influence the interaction of EVs with environmental proteins especially those bearing reactive thiol moieties^43^. Our investigation showed that DTT treatment is far from being optimal, at any temperature since DTT appears to induce the formation of scrambled SS-bonds linking non-vesicular proteins to the surface of EVs, in particular, albumin in sera and uromodulinin urine, as demonstrated by immuno-blotting. Both these proteins display a high content of Cys residues (35 in albumin^44^, 48 in uromodulin and fibrinogen 58 cyst^45^ which could promote the formation of disulphide bridges^43^.

Data clearly show that EVs contamination could be reduced using Tris as buffer and β-ME as a reducing agent, all steps better performed at T_amb_. These conditions are thus recommended in order to obtain maximum purity vesicles for biomarker investigations. As far as MVs are concerned, the presence of Tris lowered contamination also at 37 °C (Fig. 4). However, when Tris concentration was increased, regardless of the presence of β-ME, a “salting-out” effect (Fig. S3) causing a massive protein aggregation in exosomes was observed. Nanoparticle analysis confirmed that the cleanest samples, as judged by electrophoresis, are those obtained in the presence of β-ME (where single peaks are seen) after sucrose density gradient. In the absence of reducing agent or in the presence of DTT other smaller peaks are present, coming from material with different size. This is more evident in the case of MVs. Nanoparticle analysis also showed that the above-mentioned conditions lower contamination from environmental high abundance proteins: in the presence of β-ME peaks were narrower but their height was not lower (Fig. 3). This demonstrates that, while the protein concentration of samples diminishes with the use of Tris and β-ME, samples do not lose intrinsic vesicle proteins, rather they are losing contaminants. This extravesicular protein removal is also confirmed by the analysis of the supernatants remaining after centrifugation used to pellet vesicle obtained after purification.

On the other hand, in addition to disulphide bridges, other ionic and non-ionic interactions may occur on the EVs surface. It is known that electrostatic interactions on hydrophilic surfaces are one of the main factors determining the adsorption of biomolecules on particle surfaces. Another cause of protein absorption could be the zeta potential of MVs surface, that can form ionic bonds carried by Phosphate^2−^, abundant in PBS^46–48^. For these reasons, it is likely that also other less abundant proteins, present in these two biological fluids, could bind to EVs rendering the analyses less reliable and complicating the process of biomarker discovery by MS. Based on our experiment results, gel electrophoresis clearly highlights the eventual presence of smeared protein patterns, characteristic of non-vesicular proteins. As a matter of fact, even if by a quantitative point of view, MS is the perfect choice, it is both time consuming and very expensive when compared to gel electrophoresis.

Blood is a commonly used biological sample acquiring a considerable interest due to its minimally invasive mode of collection and the information it can give thanks to its content in EVs. As far as blood is concerned, it has already been suggested that additional purification steps may be necessaryto eliminate contaminants from EVs^41^. Data show that, besides their biogenesis and function, EXOs are different from MVs especially for their propensity to bind non-vesicular high abundance proteins in each physiological milieu. In particular, it appears that a key feature of EXOs is their being players of surface interactions: it has been observed that EVs may represent a uniquely large interactive surface area, and the surface interactome may play a role in different physiological and pathological processes. Data suggest that EXOs in particular have the ability to bind plasma and urine high abundance components, which may explain their recognized role in malignancies. Likely, high abundance proteins bind more to the EXOs, respect to MVs due to their relatively large surface to volume ratio. However, the same EXOs sample split in aliquots bound more environmental proteins when treated with PBS/DTT than with Tris/β-ME. On the other hand, also MVs bind extravesicular proteins in urine (see uromodulin and albumin in Figs 7 and 8), although to a lower extent than EXOs. The overall level of extravesicular proteins bound to the EXOs was too high as compared to MVs, to be merely attributed to the higher surface to volume ratio of EXOs. We cannot rule out that fact that the characteristics of the milieu, of the environmental protein and also differential specific surface features can play a role in the binding of extra-vesicular protein to the EVs surface. Further studies are needed to study the physiological relevance of the EVs surface interactions.

Contamination from non-vesicular materials is a critical issue linked to centrifugation-based methods for EVs isolation, that can cause unwanted variability to the down-stream analyses. The ability to discern contaminant from bona fide EVs proteins would allow to define the EVs proteome composition fully exploiting the EVs biomarker potential. Isolation of both EXOs and MVs from blood with minimal contamination by plasma proteins is, therefore, of great importance in the search for biomarkers for example of solid and hematologic malignancies^49,50^. Haraszti *et al*.^51^ found a multi-protein marker phenotyping tool useful in different pathologies, cancer included. As a matter of fact, cancer cells release EXOs into blood to mediate tumour-related processes and metastases. As far as urinary tract malignancies are concerned, and considered that urinary EXOs contain most of the urinoma proteome, urine can become a non-invasive “liquid biopsy”^52^. with the potential to reduce tumor biopsies and to detect new useful biomarkers^53–55^. Precision medicine represents the starting point of a new epoch not only in the management of diseases in a less invasive way but also in the discovery and use of new biomarker for a better patient stratification^56^. For example, Clark *et al*.^57^ identified 2,179 proteins in EXOs purified from healthy human serum, thus since vesicles can be detected in blood and urines of patients with various diseases, the development of a platform that exploits their use as a diagnostic tool has been proposed by many Authors.

In conclusion, improving purity of both EXOs and MVs, especially those isolated from biological fluids, allows to increase the specificity and selectivity of the source of protein identification for biomarker search in various diseases, among which tumors. In view of this, new experimental designs will be possible, no longer altered by the noise of the biological environment displaying greater adherence to the physiopathology processes.

## Methods

### Materials

Sucrose, Tris (hydroxymethyl) aminomethane (Tris), β-mercaptoethanol (β-ME), Ethylene glycol-bis (2-aminoethylether)-N,N,N′,N′-tetraacetic acid (EGTA), Dithiothreitol (DTT), Phosphate buffered saline (PBS), sodium phosphate, Tri-n-butylphosphine (TBP), Acrylamide, bis-acrylamide, Sodium dodecylsulphate (SDS), ammoniumpersulphate (APS), Tetra-methylethylene-diamine (TEMED), Glycine, Methanol, Bovine serum albumin (BSA), Tris-buffered saline (TBS) and Tween as well as all other analytical grade chemicals were from Sigma-Aldrich (St. Louis, Missouri, USA). FITC-Phalloidin was from Sigma-Aldrich (S. Luis, MO, USA.) IgG1 isotype controls (clone MOPC-21) and conjugated antibodies against CD41a (IgG1, clone HIP8), CD63 (IgG1, clone H5C6) and CD81 (IgG1, clone JS-81) were from Becton Dickinson (CA, USA). Mega-Mix beads were from BioCytex (Stago Group). CoomassieG-250, SDS, broad range molecular mass calibration kit, Protean II xi cell system, Quantity one software analysis program vs. 4.5, VersaDoc 4000 and Molecular Imager GS-800 calibrated densitometer were from BioRad (Hercules, CA, USA). Avanti™ J-25 centrifuge and Optima™ l–90 K were from Beckman (Brea, California, USA). Zetasizer Nano instrument (Malvern Instruments, Worcestershire, UK).

Complete protease inhibitor cocktail tablets were from Roche Diagnostics, (Basel, CH). Monoclonal mouse anti-human β-actin was kindly provided by MD Giulio Gabbiani, (University of Geneva, Switzerland). Polyclonal rabbit anti-human serum albumin (HSA) was purchased from Dako (Copenhagen F., Dk), Polyclonal sheep anti-human uromodulin was obtained from Abcam (Cambridge, UK), Polyclonal anti-human Apolopoprotein from NOVUS (Brussels, Belgium) and anti-rabbit and sheep peroxidase secondary antibodies were obtained from Immunological Sciences (Rome, Italy). Nitrocellulose Protean B membrane was from Whatman, (Boston, Ma, USA) while Super-Signal West Pico chemi-luminescent Substrate from Pierce, (Rockford, IL, USA).

### Sample collection and storage

Serum (10 mL), plasma (10 mL) and the second morning urine (120 mL) from healthy donors (12 individuals, age 35–50 years, 6 males and 6 females) were collected after informed consent. All samples were added with tablets of protease inhibitor immediately after collection, chilled on ice and centrifuged at 4 °C for 10 min respectively at 3,000 rpm or 1,000 × g to eliminate cell debris in accordance to Standard Protocols. The urines and sera were further centrifuged at 16,000 × g in a JA-20 rotor (Beckman Avanti J-25) for 30 min at 16 °C to remove microscopic particle sediments and mitochondrial fraction. Samples were pooled (three different pools, each consisting of two males and two females). An aliquot of 16,000 × g supernatant, after Bradford protein assay, was dialyzed three times against 25 mM sodium phosphate pH 7.2 in 3500 MWCO Spectra-Por cellulose membranes at 4 °C. Finally, supernatants were divided into several aliquots of 1 mL (serum and plasma) and 12 mL (urine) and stored at −80 °C until analysis. We obtained written approval of the protocol by the local Independent Ethics Committee (Comitato Etico Regione Liguria) on October 14, 2014 (study number: 408REG2014).

### Flow cytometry

Cytometer setting: before starting acquisition, LSRFortessa X-20 (Becton Dickinson), daily calibrated using CS&T beads (Becton Dickinson), was carefully washed with double-distilled water. According to Poncellet and Inglis^58,59^, FSC and SSC were set to log scale. Voltages were adjusted to the highest values that excluded the majority of background noise on the basis of DD water and pure ultracentrifuged (20,000 × g for 10 minutes) PBS acquisition and that allowed the detection of all different dimensions (0.5, 0.24, 0.2, 0.16 um) of SSC MegaMix beads (BioCytex, Stago Group) and Rosette Calibration beads (Exometry, NL)^60^.

Serum and plasma extracellular vesicle pellets (both MVs and Exos) were stained with Phalloidin (high-affinity filamentous actin, F-actin), CellTrace FR (Thermofisher) and either CD41a or CD81 and CD63. All fluorescent probes were ultracentrifuged (20.000 × g for 10 minutes) before using. Microvesicles were first gated on the basis of their physical dimensions then, to avoid cell debris, among CellTrace positive events, those negative for Phalloidin and positive for CD41a were chosen as possible platelet-derived MVs^40^. Exosomes, positive for CellTrace and negative for Phalloidin, were then tested for their CD81 and CD63^61^ expression. For what concern EXOs, given their small dimensions, to set the proper positivity cut off, we performed a single staining analysis^62^. All pellets were then resuspended in 300 μl of filtered PBS, acquired on LSRFortessa X-20 and analyzed using Kaluza software (Beckman Coulter).

### Dynamic light scattering analysis

The size of EXOs and MVs was determined by dynamic light scattering (DLS) using a Zetasizernano ZS90 particle sizer at a 90° fixed angle (Malvern Instruments, Worcestershire, UK). The particle diameter was calculated using the Stokes–Einstein equation. For particle sizing in solution, EXOs or MVs aliquots were diluted in 10% PBS and analyzed at a constant 25 °C. The data were acquired and analyzed using Dispersion Technology Software (Malvern Instruments).

### Isolation of serum and urinary extracellular vesicles

Extracellular vesicles were isolated from the supernatant of sera, plasma and urines. The micro-vesicles were isolated from the 16,000 × g supernatant by centrifugation at 22,000 × g in a JA-20 rotor (Beckman Avanti™ J-25) for 120 min at 16 °C. The 22,000 × g supernatant was ultracentrifuged at 100,000 × g in a Ti 90 rotor (Beckman Optima™ l–90 K) for 120 min at 16 °C to pellet the EXOs. An aliquot of each pellet was tested for protein content by Bradford protein assay. For each sample, the mean and standard deviation of the protein quantification is reported (MVs plasma 37 ± 2.6 μg/ml, serum 32 ± 2.2 μg/ml and urine 21 ± 1.5 μg/ml; EXOs 28 ± 2.0 μg/ml, serum 24 ± 1.7 μg/ml and urine 16 ± 1.1 μg/ml). Until use, all samples were stored at −80 °C. Both urinary MVs and EXOs were routinely cytofluorimetrically assayed for the expression of specific markers, as previously reported^9^.

### Density gradient centrifugation

To isolate EVs with higher purity, both 22,000 × g and 100,000 × g pellets, prior to final washings with PBS, were subjected to further centrifugation in combination with sucrose cushions. Pellets, resuspended in 1 mL of isolation solution (0.25 M sucrose in 50 mMTris density 1.018 g/ml) + 2.5% β-ME or 100 mM DTT (incubation at room temperature for 30 minutes), were loaded on 1 mL of 50 mM Tris or PBS in a 30% sucrose cushion (density 1.140 g/ml) and centrifuged at 22,000/100,000 × g for 120 min at 16 °C. This 50 mMTris buffer facilitates the solubilization of filaments and keeps uromodulin in solution. To remove remaining proteins and other contaminants, pellets were subsequently washed 3 times in 1 mL of PBS, always at 22,000/100,000 × g for 45 min at 4 °C. Bradford protein assay was also after density gradient centrifugation. Protein concentrations of MVs samples were: 30 ± 2.1, 25 ± 1.7 and 16 ± 1.1 µg/ml for plasma, serum and urine, respectively. Protein concentrations of EXOs samples were: 22 ± 1.5, 16 ± 1.2 and 11 ± 0.7 µg/ml for plasma, serum and urine, respectively. As described above, each pellet was further divided into aliquots and used for the different analyzes. Until use, all samples were stored at −80 °C.

### SDS-PAGE, staining and western blot analysis

Serum and urinary extracellular vesicle pellets were dissolved and separated by gradient Sodium Dodecyl Sulphate - PolyAcrylamide Gel Electrophoresis (SDS-PAGE) according to Laemmli^63^ in a 8–16 T% polyacrylamide gels. After SDS-PAGE, proteins were visualized by a double staining procedure, based on methyl-trichloro-acetate^64^ followed by silver staining. Stained gels were digitized by using a GS-800 calibrated densitometer at 63.5 microns of resolution and analyzed with Quantity one software (Bio-Rad). In a second set of experiments, in which the loaded samples were aligned with β-actin, the proteins were trans-blotted to nitrocellulose protean membranes with a Novablot semidry system (GE Healthcare, Milan, Italy) using a continuous buffer system (2-amino 2-idroxymethyl 1,3-propanediol, 38 mM Tris, 39 mM glycine, 0.035% SDS, and 20% methanol). The run was carried out for 2 h at 400 mA at 4 °C. Hybridisation was preceded by an overnight incubation at T_amb_ with a blocking solution of 3% (wt/vol) BSA in TBS 0.15% Tween. Incubation with primary antibodies (i.e. monoclonal mouse bona fide β-actin, polyclonal rabbit anti-human serum albumin HAS, polyclonal sheep anti-human uromodulin and polyclonal anti-human Apo-A1, Apo-E and Apo-C) diluted 1:2000 (vol/vol), was performed overnight at room temperature in 3% (wt/vol) BSA in TBST. The membrane then was washed with TBST four times, 15 min each, before of the incubation with peroxidase-conjugated mouse, rabbit or sheep anti-human diluted 1:5000 (vol/vol) in 3% (wt/vol) BSA in TBS-T for 4 h at T_amb_. The membrane then was washed four times, 15 min each, before developing the immune-blot with the Super-Signal West Pico chemi-luminescent Substrate (Pierce, Rockford, IL). Chemi-luminescence was used to detect proteins after Western blot, signals being acquired with Versa Doc 4000 (Bio-Rad).

All the previously applied methods were carried out in compliance with the relevant guidelines and regulations.

### Image analysis

Images were captured and analyzed by Quantity one software. Numbers and intensities of bands were exported for further analysis. To test the quality of each separation, for both EXO and MV, the number of bands visualized, their resolution and the presence, as contaminants, of albumin or uromodulin, Apo-A1, Apo-E or Apo-C in serum or urinary samples respectively, were taken into account. Results are representative of at least five experiments. All analyses were performed using package R software last version available at the time of experiments.

### Statistical analysis

Kruskal-Wallis test was used to assess differences between all the protocols of EXOs or MVs purification. The results were expressed as mean and standard deviation. A value of P ≤ 0.05 was considered to be statistically significant. All statistical tests were performed using the latest version of software package *R* available at the time of the experiments.

## Supplementary information

Supplementary information
