# Publisher Correction: Biological surface properties in extracellular vesicles and their effect on cargo proteins

**DOI:** 10.1038/s41598-019-54948-8

**Published:** 2019-12-05

**Authors:** Laura Santucci, Maurizio Bruschi, Genny Del Zotto, Francesca Antonini, Gian Marco Ghiggeri, Isabella Panfoli, Giovanni Candiano

**Affiliations:** 1 Laboratory of Molecular Nephrology, IRCCS Istituto Giannina Gaslini, Genoa, Italy; 2 Department of Research and Diagnostics, IRCCS Istituto Giannina Gaslini, Genoa, Italy; 3 Division of Nephrology, Dialysis, and Transplantation, Scientific Institute for Research and Health Care, IRCCS Istituto Giannina Gaslini, Genoa, Italy; 4 Department of Pharmacy-DIFAR, University of Genoa, Genoa, Italy

Correction to: *Scientific Reports* 10.1038/s41598-019-47598-3, published online 10 September 2019

In the original version of this Article, the author Genny Del Zotto was incorrectly indexed. This error has now been corrected.

