# Supplementary material for: Biological surface properties in extracellular vesicles and their effect on cargo proteins
Source: Sci Rep. 2019 Sep 10;9:13048. doi: 10.1038/s41598-019-47598-3 (PMC6736982; doi:10.1038/s41598-019-47598-3)
Supplement: Supplementary file 1 — Supplementary information [file 41598_2019_47598_MOESM1_ESM.docx]

**BIOLOGICAL SURFACE PROPERTIES IN EXTRACELLULAR VESICLES AND THEIR EFFECT ON CARGO PROTEINS.**

Laura Santucci^1^, Maurizio Bruschi^1^, Genny Del Zotto^2^, Francesca Antonini^2^, Gian Marco Ghiggeri^1,3^,Isabella Panfoli^4^ and Giovanni Candiano^1*^.

^1^Laboratory of Molecular Nephrology, IRCCS Istituto Giannina Gaslini, Genoa, Italy;

^2^Department of Research and Diagnostics, IRCCS Istituto Giannina Gaslini, Genoa, Italy;

^3^Division of Nephrology, Dialysis, and Transplantation, Scientific Institute for Research and Health Care, IRCCS Istituto Giannina Gaslini, Genoa, Italy;

^4^Department of Pharmacy-DIFAR, University of Genoa, Genoa, Italy.

**Key words**: exosomes, microvesicles, serum, urines, reducing agents.

***Corresponding Author: *Dr.* Giovanni Candiano**, PhD:

Laboratory of Molecular Nephrology, IRCCS Istituto Giannina Gaslini, Via Gerolamo Gaslini 5, Genoa, 16147, Italy.

E: giovannicandiano@gaslini.org; P +39 010 5636-3522.

**SUPPLEMENTARY FIGURE and LEGENDS**

**
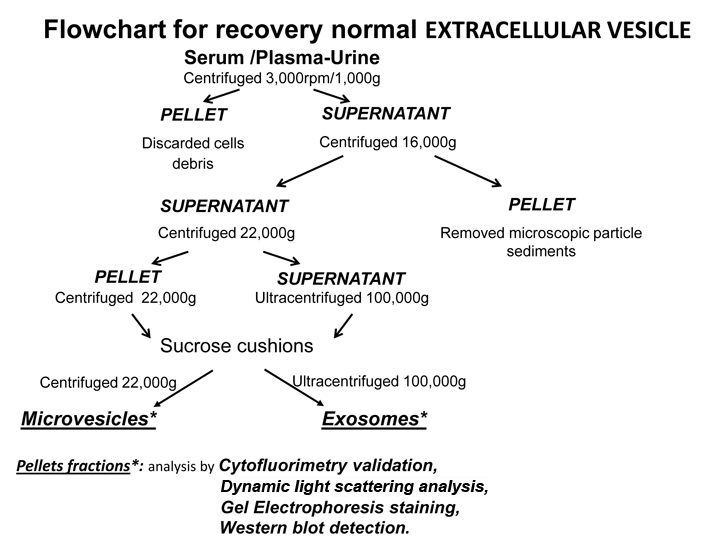
**

***Supplementary Figure S1.*** Flowchart of the procedure used to analyze the serum/urinary extracellular vesicles. Samples were centrifuged to eliminate cell debris first and subsequently to remove the microscopic particle sediments. Then, microvesicle and exosome fractions (22,000 and 100,000 × g respectively) were purified. The pellets were subjected to further centrifugation in combination with sucrose cushion to isolate the extracellular vesicles with higher purity. Each pellet obtained was divided into aliquots to be used for different analyses: cytofluorimetry validation, dynamic light scattering analysis, gel electrophoresis staining and western blot detection.


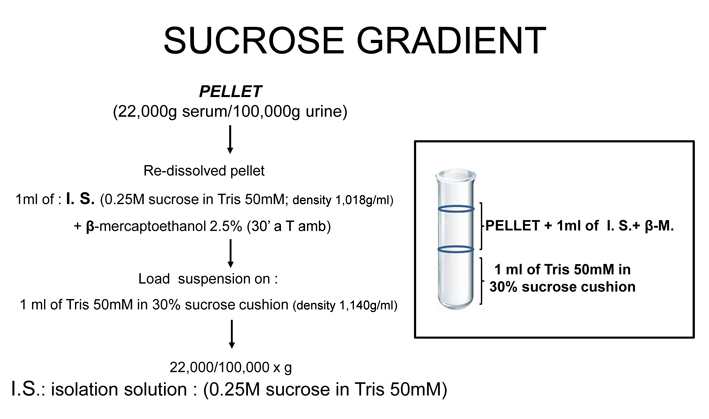


***Supplementary Figure S2.*** Flowchart of the procedure used for the density gradient centrifugation of the serum/urinary extracellular vesicles. The 22,000g and 100,000 g pellets were resuspended in 1mL of isolation solution, loaded on 30% sucrose cushion and centrifuged at 22,000/100,000 x g to obtain microvesicles and exosomes with higher purity.


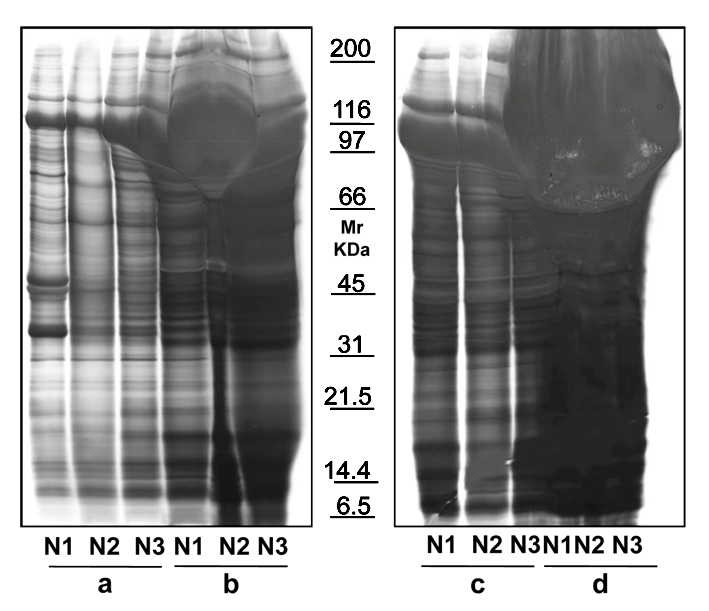


***Supplementary Figure S3.*** Representative silver staining of exosomes obtained from 1ml of three different sera (**N1-N3**) of healthy donors utilizing at increasing concentrations of Tris as buffer (50 mM in panel **a**, 250 mM in **b** and 500 mM in **c**-**d**). The samples visualized in panel **c** are treated also with the addition of reducing agent β-ME. SDS-PAGE was performed in 8-16% T gels.


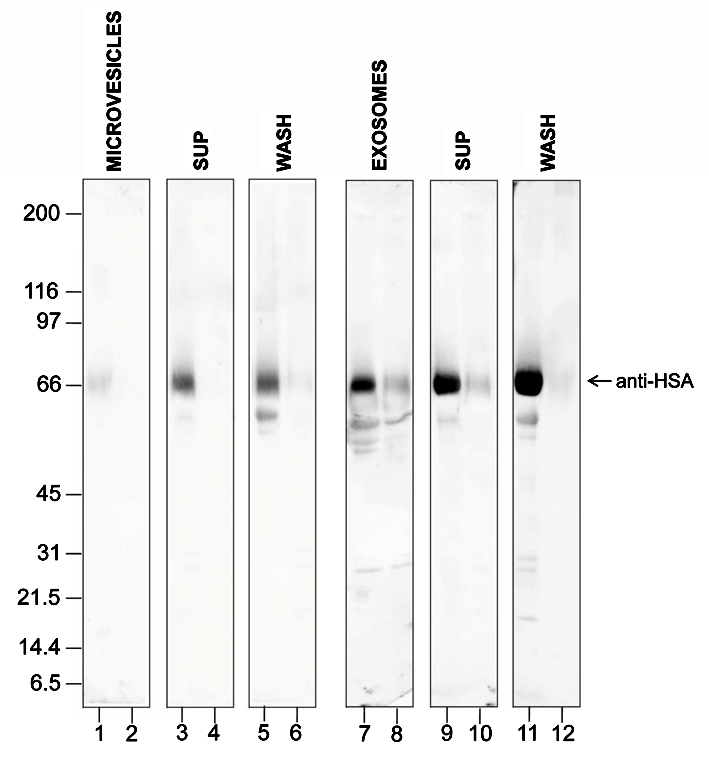


***Supplementary Figure S4.*** Representative western blot of supernatants from the first (microvesicles, lanes1, 3, 5; exosomes, lanes 7, 9, 11) and the last (microvesicles, lanes 2, 4, 6; exosomes, lanes 8, 10, 12) wash of serum from healthy donors with polyclonal rabbit serum anti-albumin (HSA). Cropped blots to better highlight the result of the protein tested. SDS-PAGE was performed in a 8-16% T gel.


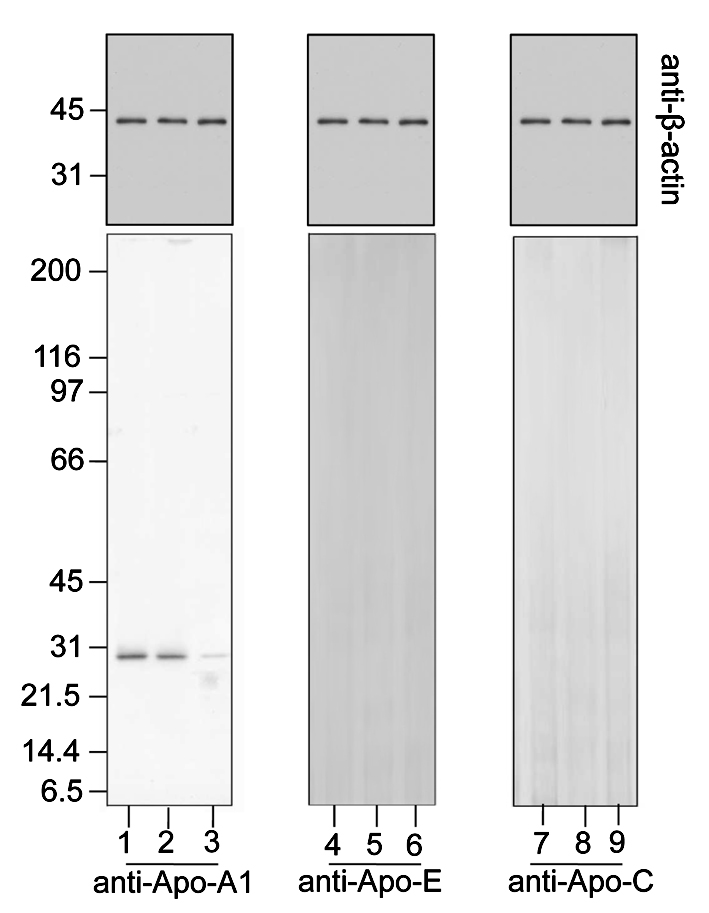


***Supplementary Figure S5.*** Representative western blot of exosomes pellets of serum from healthy donors treated without reducing agent (1, 4, 7), with DTT (2, 5, 8) and β-ME (3, 6, 9) detected by anti-human Apo-A1 or Apo-E or Apo-C on a sucrose gradient. Anti-β-actin was used as loading control. SDS-PAGE was performed on a 8-16% T gel.
